# First Trimester Abortion: A Rare Cause of Intrauterine Bony Spicules

**DOI:** 10.1155/2012/701021

**Published:** 2012-06-20

**Authors:** Anshuja Singla, Bindiya Gupta, Kiran Guleria

**Affiliations:** Department of Obstetrics & Gynaecology, University College of Medical Sciences and Guru Teg Bahadur Hospital, Dilshad Garden, Delhi 110095, India

## Abstract

Bony fragments in the uterus occur after second trimester termination of pregnancy following retained fetal bones. Very rarely, they can form following first trimester loss. Clinical symptoms range from pain, menstrual symptoms, and infertility. Ultrasound shows a hyperechoic shadow, and treatment is by curettage or hysteroscopic removal.

## 1. Case Report 

A 23-year-old woman presented with complaints of secondary infertility and postmenstrual spotting for one year. She gave a history of missed abortion of ten-week period of gestation for which she underwent dilatation and curettage for retained products after two months of abortion. Her menstrual cycles were regular with normal flow. General physical and pelvic examination was normal. 

**Figure 1:**
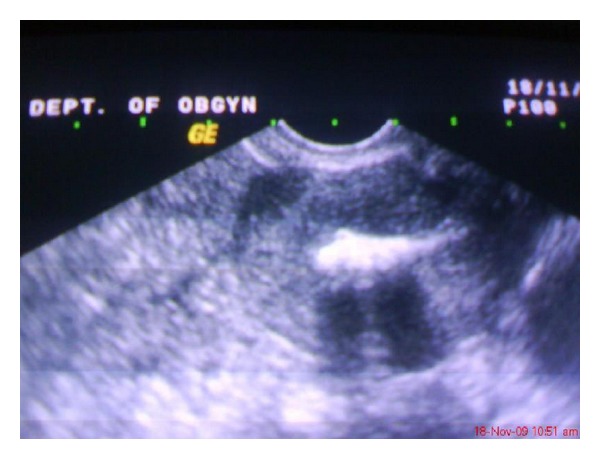
Transvaginal ultrasonography of the uterus revealed calcific areas.

**Figure 2:**
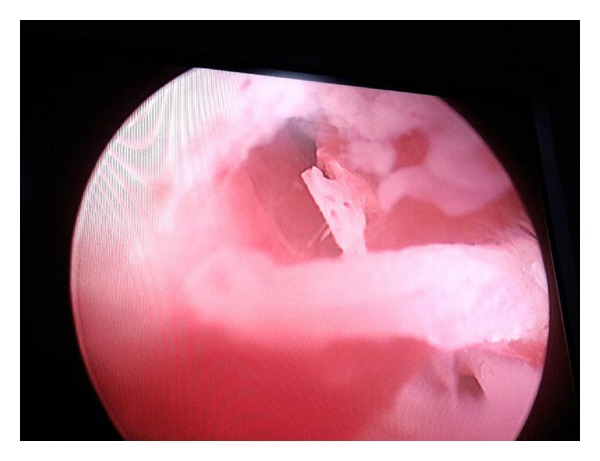
Hysteroscopic view of the uterus.

**Figure 3:**
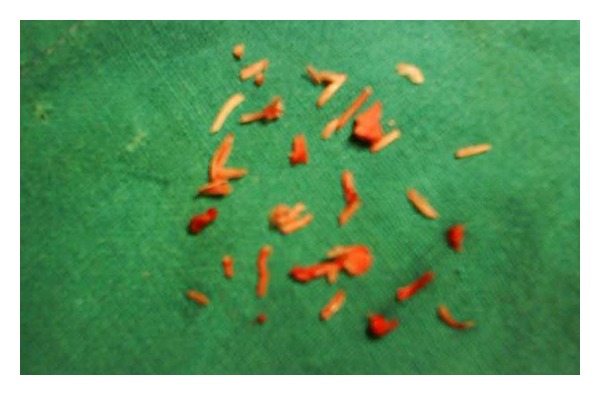
Bony fragments removed from the uterus.

Transvaginal ultrasonography revealed a linear hyperechogenic endometrial shadow, suggestive of calcified lesions (Figure 1). Diagnostic hysteroscopy revealed a pale endometrium with white, bone-like, and spicules occupying almost whole of the cavity till the internal os and extending into the posterior uterine wall (Figure 2). The cervix was dilated, and the bony spicules were removed from the uterine cavity by curettage (Figure 3). Histopathologic examination confirmed multiple bony pieces suggestive of retained product of conception.

Following this report, the history was reviewed again in which the patient confessed of having a premarital second trimester medical termination of pregnancy three years back. Three weeks after surgery although the patient had a normal menstrual period, ultrasound showed persistence of calcifications. She was posted for a repeat hysteroscopic removal of bony spicules. This time her evacuation was complete, and her follow-up scan after two weeks was normal. On followup, the patient's hormonal profile and her husband's semen analysis are within normal limits, and she has been advised a hysterosalpingogram for tubal patency. 

## 2. Discussion 

Majority of cases of endometrial ossification present with secondary infertility after second trimester abortions but symptoms of menometrorrhagia, dysmenorrhea, vaginal discharge, pelvic pain, and spontaneous expulsion of bony fragments in the menses can also be seen in some [1]. In this case, the patient had a history of both first and second trimesters termination of pregnancy. The former seems to be a more probable cause for bony fragments in the present case, as the patient had conceived following the second trimester termination. First trimester abortions are an unusual cause of fetal bones.

Occasionally dystrophic calcification and ossification of retained fetal tissue may result in bone formation [2]. Besides this, osseous metaplasia of mature endometrial stromal cells due to prolonged chronic inflammation and tissue destruction can also result in heterotopic bone formation in the uterus [3]. Our patient presented both with menstrual complaints and infertility. 

Endometrial ossification may cause secondary infertility by three mechanisms: (1) prevention of implantation as a result of obliteration of the uterine cavity (mechanical effect); (2) prevention of implantation as a result of the chronic inflammatory effect of intrauterine bony fragments (intrauterine device like effect); (3) direct toxicity of osseous particles on the embryo (embryo toxicity) [4]. It has been shown that the removal of these fragments reduces the local concentration of prostaglandins in 15% [5]. The diagnosis is suspected at ultrasound and confirmed on hysteroscopy and histopathologic examination. Treatment is by ultrasound guided curettage or by hysteroscopic removal [6]. Besides retained bones, other causes of calcification, namely, tuberculosis, foreign body, and copper-T also need to be ruled out. 

## 3. Conclusion 

To conclude, bony fragments in the uterus can be a possible cause of infertility especially following medical termination of pregnancy and can even occur after first trimester abortion. A strong possibility should be kept when ultrasound shows hyperechoic endometrium, and removal of bony fragments by hysteroscopy and curettage is associated with therapeutic success.
